# Reliability of Measurements of Rat Lateral Gastrocnemius Architectural Parameters Obtained from Ultrasound Biomicroscopic Images

**DOI:** 10.1371/journal.pone.0087691

**Published:** 2014-02-05

**Authors:** Carolina Carneiro Peixinho, Natália Santos da Fonseca Martins, Liliam Fernandes de Oliveira, João Carlos Machado

**Affiliations:** 1 Biomedical Engineering Program, COPPE, Federal University of Rio de Janeiro, Rio de Janeiro, RJ, Brazil; 2 Laboratory of Biomechanics, Department of Biosciences and Physical Activity, Federal University of Rio de Janeiro, Rio de Janeiro, RJ, Brazil; 3 Post-Graduation Program in Surgical Sciences, Department of Surgery, School of Medicine, Federal University of Rio de Janeiro, Rio de Janeiro, RJ, Brazil; Faculty of Animal Sciences and Food Engineering, University of São Paulo, Pirassununga, SP, Brazil, Brazil

## Abstract

This study used ultrasound biomicroscopy (UBM) to quantify the pennation angle (PA) and muscle thickness (MT) of rat skeletal muscle and evaluated the reliability and reproducibility of the method by statistical analysis, determining the coefficient of variation (CV), intraclass correlation coefficient (ICC) and typical error of measurement. A UBM system with a center frequency of 40 MHz was used to acquire images of the right lateral gastrocnemius of ten male *Wistar* rats on two different days and with two ankle positions (90° or 150°). Two independent measurements of the PA and MT were randomly performed in each of three picture frames. The analysis resulted in CVs of 10.47% and 4.81% for the PA and the MT, respectively, for the ankle at 90° and 9.24% and 5.98% for the ankle at 150°. Additionally, the ICC values ranged from 0.75 to 0.92 for the PA and 0.57 to 0.99 for the MT. Statistically significant differences between the ankle positions were observed for the PA (p = 0.00013). The reliability of the PA and MT measurements for the rat right lateral gastrocnemius, determined from the ultrasound biomicroscopy images, was high (>0.90) for the methodology proposed. This finding indicates the potential of ultrasound biomicroscopy for quantitative muscle characterization and the longitudinal examination of tissue adaptation to different conditions of use, disease and rehabilitation.

## Introduction

Ultrasound imaging is widely used to monitor changes in and adaptations of the skeletal muscle submitted to different conditions [1], [2], [3]. In small-animal models, high-frequency ultrasound can generate scalable images to follow-up morphological processes, allowing the investigation of the influence of different injuries, diseases and training programs on skeletal muscle, especially of those interventions which may not be performed or strictly controlled in humans. Ultrasound biomicroscopy (UBM) generates such high-resolution images, providing structural information and facilitating *in vivo* longitudinal studies. The UBM frequencies used in most applications (40 to 60 MHz) correspond to a spatial resolution on the order of tens of micrometers, which is suitable for imaging small-animal muscle tissue with a pattern similar to that of images acquired by applying conventional ultrasound to human tissue [1], [2], [3]. The importance of a longitudinal follow-up for different processes, helping to minimize animal losses during long-term studies and to obtain more reliable information, has been highlighted by other authors [4]. Peixinho et al. [5] recently described the accompaniment of the skeletal muscle regeneration process, suggesting that the UBM technique is adequate to be implemented in future studies with treatments, such as experimental drugs, that must be assessed in animals before progressing to humans.

The structural characteristics of the muscle fascicles, such as the pennation angle (PA), fiber length (FL), muscle thickness (MT) and physiological cross-sectional area (PCSA), may be correlated with muscle function [6]. These parameters are commonly assessed in studies of the skeletal muscle because functional demands lead to a rearrangement of the muscle architecture, affecting the maximum muscle force and contraction speed [6]–[13]. Such studies demonstrate the *in vivo* changes in the muscle architecture due to the joint angle and passive or active contraction conditions [10]–[12]. Additionally, muscle plasticity, when examined in different experimental models of increased use or disuse, has also been reported in analyses of human subjects [7], [8], [13]. However, the reliability and validity of the ultrasound imaging-based quantification of the parameters pertaining to muscle architecture are often reported only by coefficients of variation [14]; and important information as the typical error of measurement (TEM) and intraclass correlation coefficient (ICC) are scarcely presented. Differences between muscles and methodologies indicate the need of further reliability investigations.

Regarding animal studies, data related to muscle architecture have been obtained *in vitro* for certain rodents, including rats. Eng *et al*. [15] examined the muscle architecture and fiber type of the rat hindlimb to define the functional specialization of each muscle, whereas Witte *et al.* [16] used UBM to describe the coordination of individual fibers of rat and mouse muscle by *ex vivo* testing. UBM has also been used to quantify the architectural parameters of rat skeletal muscle *in vivo* following muscle adaptations to an injury [5]. In this case, a preliminary analysis of the variability and reliability of the measurements, and more specifically, of the muscle thickness and pennation angle, was performed. A more recent study assessed the inter-rater reliability of the measurements of rat gastrocnemius muscle thickness, although this work used conventional ultrasound images [4].

Similar to any quantitative investigation, the evaluation of muscle plasticity based on the architectural parameters obtained using an imaging method must also consider the measurement errors inherent in the image analysis protocol. The knowledge of measurement errors is essential to the accurate assessment of muscle function, force and adaptations to different conditions of use, disease and rehabilitation. Among several metrics for error measurement, the TEM and the ICC can reveal outcome differences according to the adopted measurement protocol (inter-observer, inter-day or inter-images). The reliability of a muscle architecture parameter determined from an ultrasound image can be affected by the machine operator’s skill and the presence of intervening tissues, such as a fat layer over the muscle or vessels, which can hinder the identification of the architectural parameter and, consequently, the measurement quality. A few of the major limitations of determining muscle architecture parameters using ultrasound images are a lack of clear identification of the tissue interfaces (fat-muscle, connective tissue-muscle tissue and muscle-bone) and of the regions of interest, which could lead to significant measurement errors. An additional difficulty inherent to UBM is the relative positioning of the transducer and the animal’s hindlimb, Due to the large probe size, its positioning cannot be as precise (aided by anatomical markers) as in human experiments which could add errors to the measures.

For practical purposes, measured results should be reported in full, including the method and results of the reliability analysis. This protocol will allow a comparison with results obtained elsewhere and legitimize, for example, findings related to the tracking of muscle adaptations in animal models of muscle injuries and treatments.

The present study was designed to assess the reliability of measurements of two architectural parameters, pennation angle and muscle thickness, obtained from ultrasound biomicroscopic images of rat lateral gastrocnemius (LG) muscles. The reliability of the results was evaluated by considering the calculation of the architectural parameters and digitizing procedures, including the choice of measurement sites, image acquisition and digitization. The same experimental method was tested for two ankle positions: 90° and 150°.

## Materials and Methods

### A. Ethics Statements

All of the experimental protocols were performed in compliance with the recommendations of and approved by the Institutional Care and Animal Use Committee of Federal University of Rio de Janeiro (Permit number: 01/11).

### B. Animals

**Figure 1:**
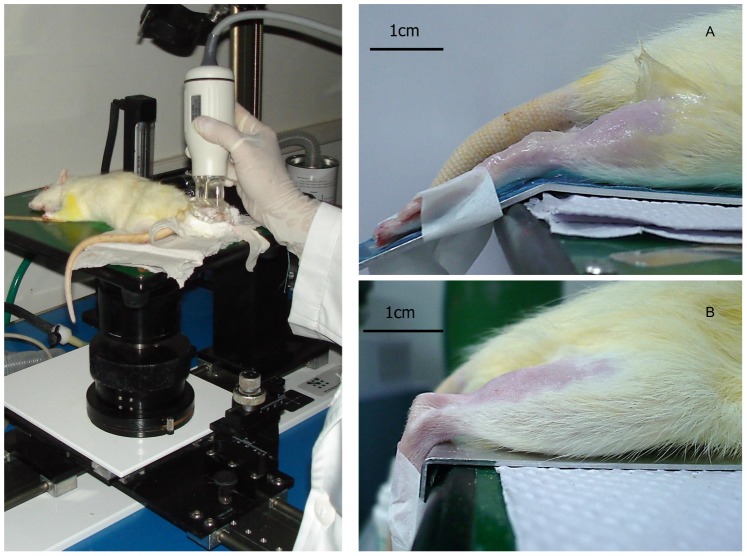
Rat hindlimb immobilization for image acquisition. Picture of a rat hindlimb immobilized for image acquisition with the aid of an external fixation at (A) 150° and (B) 90°.

The right LG muscles of ten male *Wistar* rats (3–4 months, 200–250 g) were scanned using a UBM machine, with ankle angles of 90° and 150° (Fig. 1) imposed by the use of an external fixation. All of the animals were assessed on two different days within an interval of one week.

### C. Procedures

An UBM imaging system (Vevo 770; VisualSonics, Toronto, Canada) with a center frequency of 40 MHz, sampling frequency of 34 Hz, and lateral and axial resolutions of 80 and 40 µm respectively, was used.

Prior to image acquisition, the rats were anesthetized with an intraperitoneal injection of ketamine (10–15 mg/kg) and xylazine (50–75 mg/kg), and their legs were tricotomized. The rats were then positioned in ventral decubitus on the equipment platform, and their ankles were randomly immobilized at 90° or 150°, with the posterior leg free to be assessed using a UBM probe. Longitudinal image planes, related to the segment’s longitudinal axis, were acquired from each rat by the same machine operator and for both ankle positions. The probe was positioned over the posterior leg and manual mobilization of it was performed until LG was clearly visualized. Minimal probe displacements away from the exact position resulted in poor-quality images, due to the high frequencies used that are associated with high resolution. The images of the LG were obtained with the muscle positioned in the depth of field of the ultrasound beam, and only those images with sufficient fiber distinction were saved for further analysis.

Ultrasound gel coupled the probe to the animals’ skin. Minimal pressure was applied to the muscle because the transducer did not directly touch the skin, minimizing errors in muscle thickness and fascicle angle measurements.

An evaluator with experience in ultrasonographic measurements determined the PA and MT of the most visually identifiable fibers in the high-resolution US images. Image processing software (ImageJ; National Institutes of Health, Bethesda, Maryland, USA) was used to perform the measurements after adjusting an image’s grayscale and magnification levels, allowing the muscle structures to be better visualized.

**Figure 2:**
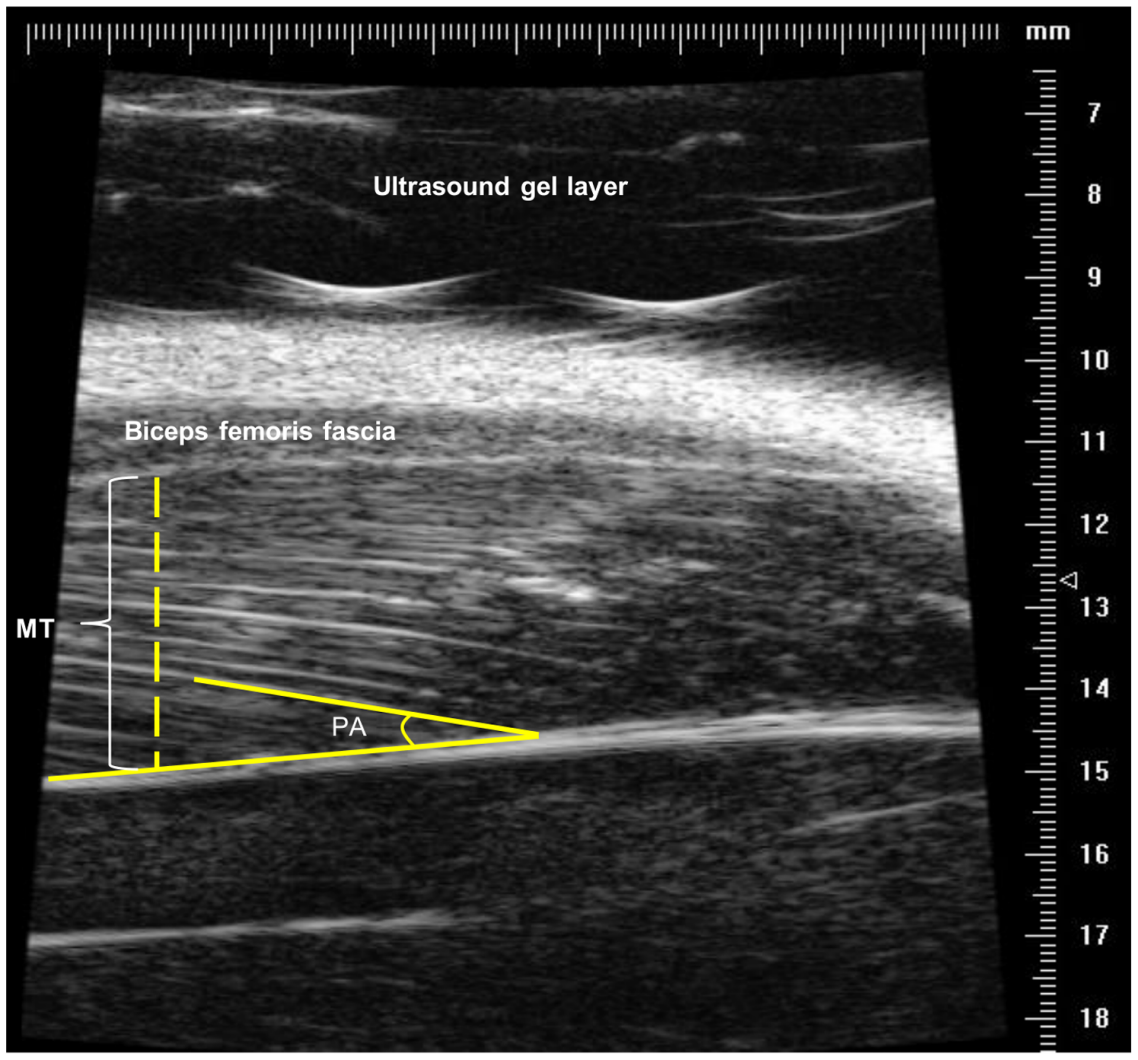
Quantification of muscle thickness and pennation angle on the UBM image. UBM image showing muscle details and the traces used to quantify the muscle thickness (MT) and pennation angle (PA) of the lateral gastrocnemius muscle.

A typical longitudinal UBM image of the rat hindlimb is depicted in Fig. 2, with a clear delineation of the muscle structures and a proper definition of the aponeuroses and fascicles used to determine the selected muscle architectural parameters. The MT was defined as the distance between the superficial and the deep aponeuroses, considering the left side of the UBM image as the region of interest, which corresponded to the maximum value of the MT, according to previous analysis of several animals. A combination of UBM high resolution, probe field of view, beam scanned and LG muscle size requires a confined exactly probe position to acquire muscle image with maximal muscle thickness. Moreover, the PA was calculated from the positive angle between the deep aponeurosis and the line of the fascicle.

Three image frames were selected from each video recorded by the UBM equipment for each animal in each ankle position and each test day. The criterion for frame selection was that the fascicles and aponeuroses were best distinguished among the images, according to the possibility of quantification of the parameters. In each of the three frames, two independent measurements of both parameters were randomly performed by the evaluator, with a total of six measurements of PA and six of MT per rat for each of the ankle positions for each test day. Considering the measurements of ten rats obtained on two different days, a total of 480 values of PA and MT were obtained.

### D. Statistical Analysis

A statistical analysis was conducted using the software STATISTIC 7.0 (StatSoft; Oakland, USA). The tests analyzed the data dispersion and reliability and also compared the measured values from two different days of image acquisition, and the values corresponding to the different ankle positions. The normal distribution of the data was tested using the Shapiro-Wilk test. Coefficients of variation were calculated for both parameters (PA and MT) and the two ankle positions, as well as for the two days of image acquisition. Two-way ANOVA with repeated measures was used to analyze the differences in the PA and MT data measured twice from the three selected frames (image×measure). F-ratios were considered significant at p<0.05. Significant differences between means, at p<0.05, were detected using Tukey’s post-hoc test. To compare the results obtained on two different days and for the two ankle positions (days×ankle position), statistical tests for dependent samples were applied (t-test and Wilcoxon matched pair test, respectively). To determine the reproducibility of the architectural parameters, the intraclass correlation coefficient (ICC), TEM and coefficient of variation (CV) were obtained according to procedures described by Hopkins [17]. The level of statistical significance was set at p<0.05 for all of the tests.

## Results

The statistical analysis revealed no differences between the two random measurements of each parameter (PA and MT) that were performed in each of the three frames. Therefore, the mean values were used in further testing. T-tests demonstrated that there were no significant differences between each of the architectural parameters determined from the images acquired on two different days. Mean values were used to compare the different ankle positions and a statistically significant difference (p = 0.00013) was found between the measured PAs at these positions (90° and 150°). MT presented no statistical difference between the two ankle positions.

The mean, SD, CV, TEM and ICC of the PA and MT values determined on each day and for each ankle position are presented in Tables 1 and 2. Tables 3 and 4 list the TEM and ICC for the differences between the measurements determined from each frame and between frames for the two ankle positions. In brief, CVs varied from 2.90 to 5.98% and from 6.87 to 10.47% for muscle thickness and pennation angle, respectively, whereas ICC ranged from 0.58 to 0.87 and from 0.75 to 0.81 for these two parameters. All ICC values were in the range of high reliability, except for the values obtained inter-days. TEM were low for all the conditions assessed.

**Table 1:** Mean, standard deviation (SD), coefficient of variation (CV), typical error of measurement (TEM) and intraclass correlation coefficient (ICC) of the pennation angle acquired on two separate days (day 1 and day 2) for the ankle positions of 90° and 150° and considering the measurements performed on both days.

	Day 1		Day 2		Both days	
	90°	150°	90°	150°	90°	150°
Mean (°)	10.556	13.417	10.069	12.692	10.349	13.081
SD (°)	1.000	1.044	0.933	0.873	1.084	1.209
CV (%)	9.472	7.778	9.268	6.874	10.475	9.244
TEM (°)					0.811	1.196
ICC					0.813	0.755

**Table 2:** Mean, standard deviation (SD), coefficient of variation (CV), typical error of measurement (TEM) and intraclass correlation coefficient (ICC) of the muscle thickness acquired on two separate days (day 1 and day 2) for the ankle positions of 90° and 150° and considering the measurements performed on both days.

	Day 1		Day 2		Both days	
	90°	150°	90°	150°	90°	150°
Mean (mm)	3.661	3.894	3.650	3.875	3.656	3.885
SD (mm)	0.106	0.118	0.134	0.138	0.176	0.232
CV (%)	2.904	3.040	3.675	3.555	4.817	5.983
TEM (mm)					0.191	0.271
ICC					0.576	0.668

**Table 3:** Typical error of measurement (TEM) and intraclass correlation coefficient (ICC) values for the ankle at 90° and 150°, considering both the two measurements of the PA determined from each of the three frames and the measurements’mean of each of the three frames compared pair-to-pair.

	90°		150°	
	TEM (°)	ICC	TEM (°)	ICC
Measurementsfrom frame 1	0.644	0.906	0.832	0.920
Measurementsfrom frame 2	0.689	0.903	0.969	0.948
Measurementsfrom frame 3	0.751	0.909	0.681	0.915
**Mean for the three frames**	**0.695**	**0.906**	**0.827**	**0.928**
Frame 1×frame 2	0.848	0.847	0.996	0.822
Frame 1×frame 3	1.211	0.595	0.907	0.778
Frame 2×frame 3	0.852	0.836	1.019	0.795
**Mean for the three** **frame pairs**	**0.970**	**0.759**	**0.974**	**0.798**

**Table 4:** Typical error of measurement (TEM) and intraclass correlation coefficient (ICC) values for an ankle at 90° and 150°, considering the two measurements of the MT determined from each of the three frames and the measurements’mean of each of the three frames compared pair-to-pair.

	90°		150°	
	TEM (mm)	ICC	TEM (mm)	ICC
Measurements fromframe 1	0.038	0.976	0.030	0.997
Measurements fromframe 2	0.044	0.968	0.054	0.983
Measurements fromframe 3	0.100	0.858	0.065	0.986
**Mean for the three frames**	**0.061**	**0.934**	**0.050**	**0.989**
Frame 1×frame 2	0.100	0.804	0.157	0.895
Frame 1×frame 3	0.145	0.771	0.169	0.901
Frame 2×frame 3	0.113	0.787	0.144	0.917
**Mean for the three** **frame pairs**	**0.119**	**0.787**	**0.156**	**0.904**

## Discussion

Eng *et al.* [15] examined the muscle architectural properties of the rat hindlimb, using *ex vivo* techniques to characterize functional specialization. The authors measured the surface pennation angle using a standard goniometer after dissecting the specimens, with the hip and knee maintained at 90° and the ankle in a neutral position, and determined a value of 14.2±3.6° for the LG. The differences between the results published by Eng *et al.* [15] and the *in vivo* findings obtained in the present study may be explained by the disparate *in vitro* and *in vivo* approaches used. More specifically, *in vitro*, *rigor mortis* may provoke a slow contraction of the muscle fibers or connective tissue contraction, and/or a reduction in muscle length may occur during the fixation process, and the position at which the muscles are fixed may vary [18]. Eng *et al.* [15] fixed the muscles with the ankle in a neutral position and with knee and hip in different positions, and it has been reported that the positioning of involved joints modifies the pennation angle [11], [19]–[21].

Strict methods were used in the present study to minimize the measurement errors, including the double-digitizing of all of the images, digitization by a single operator and operation of the UBM machine by the same individual to remove multiple-operator variation. There were no significant differences between the independent measurements performed for each image, between the different images or between distinct days of image acquisition. The coefficient of variation was 10.47% and 4.81% for the PA and the MT, respectively, for an ankle at 90° and 9.24% and 5.98% for an ankle at 150°, considering the images acquired on two different days.

These results are similar to the data previously reported by Peixinho *et al.* [5], who used UBM for the biomechanical characterization of rats with healthy or injured triceps surae. The authors performed a variability analysis on a group of rats and presented coefficients of variation of 9.37% and 3.97% for the PA and the MT, respectively, for an ankle at full extension and 15.41% and 4.99% for an ankle in a neutral position. These results and the methodology employed are similar to the findings and design of the present study, with a slight difference in the CV values for the PA for an ankle in a neutral position. This dissimilarity may be explained by the fact that the authors of the previous work did not use an external fixation for limb immobilization, thus potentially increasing the measurement variability due to ankle angle differences.

A recent study, which reported MT measurements obtained from ultrasound images of rat skeletal muscle submitted to denervation, calculated ICCs for the MT values obtained by different raters without analyzing the reproducibility for different images or days of acquisition [4]. Moreover, the authors’ method of acquiring images could not guarantee the leg position, which can influence the results because the knee and ankle joint positions are not controlled. Nevertheless, the study yielded excellent ICC values (0.84 to 0.99), and the authors emphasized the importance of the rater’s training in producing reliable measurements, which is confirmed by the present study. In addition to training, correct animal and probe positioning were also critical factors in high-quality image acquisition.

Most studies of human muscle report higher CV values than described above. Blazevich *et al.* [22] reported CVs of approximately 18% and 30% for measurements of the MT and the PA, respectively, of the human vastus lateralis using conventional US imaging. Additionally, Ito *et al*. [23] reported a CV of 7% for the PA of the human tibialis anterior. However, a study conducted by Legerlotz *et al.* [14], which used a protocol similar to that of the present study, achieved CV values ranging from 4.6% to 6% for the PA of the medial gastrocnemius, considering two joint positions (90° and maximum plantar flexion) in children aged 4–10 years and using two ultrasound machines.

Although the ICC results for both days were low for the MT, the ICC values for the same day were high (>0.90). Thus, the error of measurement should not be solely attributed to the software-based measurement procedure but also to the relative positioning between the limb and the transducer during image acquisition, which may have varied between days, increasing the chances of error. This methodological error inherent to acquisitions performed on different days should be considered in studies involving rats because it is not possible to prepare a mold (paper) on the first visit that marks anatomical points, ensuring that the transducer position and image acquisition are the same in future tests. This practice is common in studies of humans, but due to the size of the UBM transducer compared with the hindlimb of small animals, it is not possible in animal studies. Martins *et al.* [24] reported the same trend of lower ICC values when analyzing two days of tests in a study of the reliability of ultrasound measurements of the architectural parameters GM and GL, consistent with our findings. Nevertheless, the TEM and CV values obtained in the present study were lower for both the PA and the MT on both days (less than 6% for the MT and 10% for the PA; Tables 1 and 2). This phenomenon should be considered when evaluating muscle adaptations induced by increased or decreased use.

Human studies have demonstrated that the PA and MT are highly correlated with the joint position. Maganaris *et al.* [11] reported that as the ankle angle varied from 75° to 120°, the medial gastrocnemius pennation angle varied from 6° to 12° at rest and from 9° to 16° at maximum voluntary contraction, whereas the muscle thickness did not differ significantly between ankle positions. In another study, Narici *et al.* [20] reported an increase of 15.8° to 27.7° in the PA of the medial gastrocnemius, whereas the ankle angle varied from 90° to 150° without myoelectric activity. The authors concluded that the medial gastrocnemius architecture is significantly affected by changes in the joint angle when the joint is at rest. In the present work, this relationship was studied, revealing an increase in the PA from 10.36° ±1.36° to 12.43° ±1.32° (p<0,05) and in the MT from 3.64±0.08 to 3.84±0.12 mm (no significant difference) for an ankle joint angle varying from 90° to 150°, respectively. These findings are similar to the data presented by the only previous report on UBM images of rat skeletal muscle [5], which found an increase in the PA from 9.69° ±1.49° to 16.17° ±1.52° (p<0.05) and in the MT from 2.79±0.14 to 3.13±0.12 mm (p<0.05) for an ankle joint angle varying from 104.86° ±3.13° to 151.8° ±2.60°, respectively.

The reliability of the PA and MT measurements was high when repeatedly acquiring and digitizing images using the proposed UBM-based methodology, as indicated by high intra-day ICC values (>0.90) and low TEM values. This work indicates the potential of using the UBM technique for qualitative and quantitative muscle characterization, even when the muscle tissue is subjected to conditions of increased or decreased use. Further work could be performed to evaluate multi-observer variability, and a validation of this method through comparisons with direct anatomical or calibrating phantom measurements should be conducted. Additional studies in rats using UBM will also allow a longitudinal follow-up for injuries and diseases, facilitating differentiation between healthy and injured muscles at various stages after injury, and evaluating the outcomes of treatment options that must first be investigated in animals. Examples of the applicability of UBM images in longitudinal studies are the use of the methodology described to evaluate the effects of different drug treatments of muscle injuries in rats or to monitor the regeneration of a denervated muscle after a reconstructing technique, as suggested by the initial approach of Nijhuis *et al*. [4].
